# A DFT Study of CO, H_2_, C_2_H_2_ and CH_4_ Adsorption onto SnS_2_-Based Monolayers: Favorable Sensitivity and Selectivity by Doping Single Pd or Pt Atoms

**DOI:** 10.3390/molecules31122062

**Published:** 2026-06-12

**Authors:** Wenming Cheng, Hao Pan, Yuxing Zhang, Jiaming Ni

**Affiliations:** 1 School of Automotive and Transportation, Shenzhen Polytechnic University, Shenzhen 518055, China; panhao@szpu.edu.cn; 2 School of Aircraft Engineering, Nanchang Hangkong University, Nanchang 330063, China; z3103785740@gmail.com; 3 School of Materials Science and Engineering, Nanchang Hangkong University, Nanchang 330063, China; nijiaming@nchu.edu.cn

**Keywords:** first principles, SnS_2_, noble metal, gas adsorption, electric structure, recovery time

## Abstract

This study applied density functional theory (DFT) to investigate gas-sensitive devices based on Pt- and Pd-doped SnS_2_ monolayers, exploring their adsorption and sensing performance on four characteristic gases generated under normal operating or fault conditions of transformer oil. The adsorption behaviors and underlying sensing mechanisms of four gases on pristine and modified SnS_2_ were systematically elucidated. The results reveal that Pt/Pd incorporation triggers a transition from weak physisorption to robust chemisorption. Compared to intrinsic SnS_2_, the decorated monolayers exhibit dramatically augmented adsorption energies and accelerated interfacial charge transfer for all target molecules. Crucially, noble metal modification fundamentally modulates the electronic structure of the SnS_2_ lattice, endowing the material with exceptional recognition specificity for distinguishing different gas species. These theoretical insights establish Pt- and Pd-SnS_2_ as highly promising candidates for advanced DGA sensors, providing a robust materials design strategy for the condition monitoring of critical electrical infrastructure.

## 1. Introduction

Oil-immersed power transformers account for over 90% of all transformer types and serve as core equipment within high-voltage power systems [1,2]. The stable operation of transformers is crucial for ensuring the safety, reliability, power supply quality, and economic efficiency of power systems [3,4,5,6,7,8,9]. However, during prolonged operation, defects often arise due to aging insulation materials, such as localized overheating and discharge faults. These conditions can lead to degraded equipment performance and may even trigger major incidents including oil leakage, fires, and regional power outages. Research indicates that such faults catalyze the cracking of transformer oil, generating hydrocarbon gases including hydrogen (H_2_), methane (CH_4_), ethylene (C_2_H_4_), and acetylene (C_2_H_2_), alongside carbon oxides such as carbon monoxide (CO) and carbon dioxide (CO_2_), which exist in a dissolved state within the oil [10]. Consequently, Dissolved Gas Analysis (DGA) of transformer oil has gained widespread recognition as a viable and efficient online monitoring method [11]. Since the discovery of graphene—a two-dimensional layered material—its unique properties have attracted extensive attention, with graphene-based two-dimensional materials emerging as a prominent research focus [12,13,14,15,16]. Metal sulfides, as a class of materials possessing a layered structure similar to graphene, exhibit excellent chemical stability due to strong in-plane bonding within layers and weak interlayer van der Waals forces. Among these, SnS_2_, a typical IV-VI-group metal sulfide, is insoluble in water. The in-layer sulfur and tin atoms form robust covalent bonds, and the absence of dangling bonds on the surface further enhances its stability and application potential [17]. Shan et al. [18] investigated NO_2_ adsorption on SnS_2_, demonstrating favorable adsorption performance towards this gas molecule. Jin et al. [19] studied the adsorption of CO and other gases on SnS_2_, revealing weaker adsorption of CO and SO_2_ on pristine SnS_2_ surfaces, while NO, NO_2_, and NH_3_ exhibited stronger adsorption capabilities. Transition metal elements commonly used for doping include platinum (Pt), palladium (Pd), nickel (Ni), gold (Au), and silver (Ag) [20,21]. Wang et al. [22] optimized the band structure of single-layer zinc oxide by introducing Rh single atoms, substantially enhancing its adsorption capacity for NO_2_ and O_3_.

In this study, we focus on the modification effects of Pd/Pt on the monolayer structure of SnS_2_ and systematically investigate their doping stability and electronic property modulation mechanisms. The effect of these metal atoms on the adsorption of gas molecules is analyzed in depth by calculating parameters such as adsorption structure, adsorption energy, energy band structure, the density of states and recovery time.

## 2. Results and Discussion

**Figure 1:**
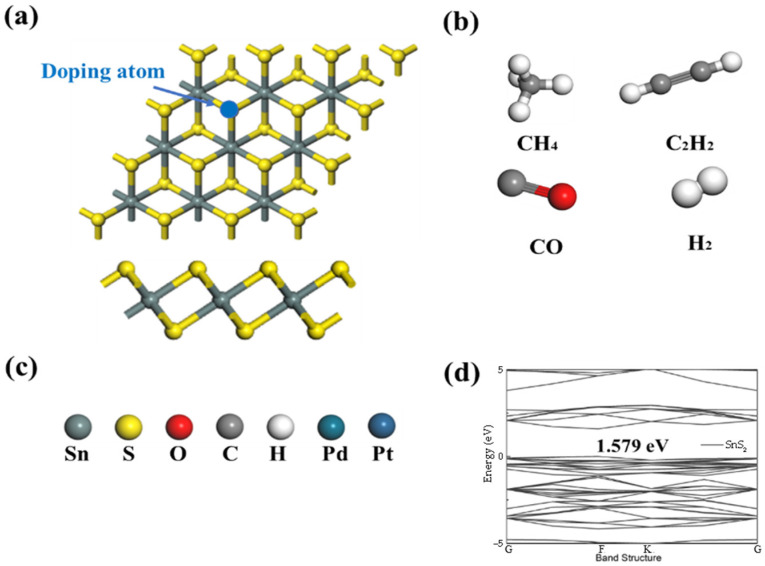
(**a**) Front and side views of SnS_2_, (**b**) the gas molecules, (**c**) the color of the atom, and (**d**) the band structure of SnS_2_.

Figure 1 shows the geometrically optimized structures of SnS_2_, its gas molecules and its energy band diagrams. It also shows the replacement of one S atom with one Pd or Pt atom in a 4 × 4 × 1 SnS_2_ monolayer supercell. In order to avoid interactions between material layers, a 20 Å thick vacuum layer is used. The bond lengths, bond angles, and lattice constants of the geometries are shown in Figure 1a,b, and the lattice constants of the optimized intrinsic SnS_2_ were all 3.34 Å; the S-Sn bond length of the pure SnS_2_ is 2.546 Å, and these calculations are in good agreement with the literature [23].

As shown in Table 1, to quantitatively evaluate the gas–adsorbent interaction and electronic coupling, we calculated the adsorption energy (E_ads_), charge transfer (Q_t_), and interatomic distance (D) for C_2_H_2_, CH_4_, CO, and H_2_ adsorbed on pristine SnS_2_, Pd–SnS_2_, and Pt–SnS_2_ monolayers, as summarized in Table 1. For pristine SnS_2_, the adsorption energies are near zero (−0.007 to −0.027 eV), indicating weak physisorption with negligible charge transfer and large intermolecular distances (0.892–2.918 Å), consistent with its inert surface response. In contrast, both Pd–SnS_2_ and Pt–SnS_2_ exhibit significantly enhanced adsorption strengths, with E_ads_ values ranging from −0.521 to −1.246 eV for Pd–SnS_2_ and –0.099 to −0.866 eV for Pt–SnS_2_, reflecting strong chemisorption behavior. Notably, Pd–SnS_2_ shows the highest adsorption energy for CO (−1.246 eV), suggesting preferential binding for this gas. Charge transfer analysis reveals that electrons flow predominantly from the gas molecules to the metal-doped surfaces (Q_t_ < 0), with Pd–SnS_2_ and Pt–SnS_2_ exhibiting the largest charge transfer for C_2_H_2_ (−0.260 e) and CO (−0.290 e), respectively. The interatomic distances (D) are substantially reduced upon doping (1.387–2.130 Å and 0.892–2.918 Å for pristine SnS_2_), indicating stronger orbital hybridization and closer contact. These results collectively demonstrate that metal doping significantly enhances gas adsorption affinity, modulates charge redistribution, and facilitates charge transfer, which are all key prerequisites for high-performance gas-sensing applications.

**Figure 2:**
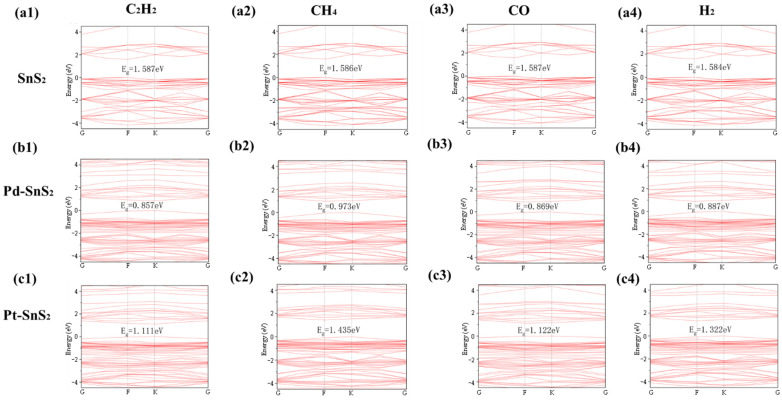
The band structure of C_2_H_2_, CH_4_, CO, and H_2_ gas molecule adsorption on pure SnS_2_ monolayers and Pd or Pt/SnS_2_ monolayers.

To elucidate the electronic structure evolution induced by metal doping and gas adsorption, we investigate the band structures of pristine SnS_2_, Pd–SnS_2_, and Pt–SnS_2_ monolayers in the presence of four representative dissolved gases (C_2_H_2_, CH_4_, CO, and H_2_) in transformer oil, as depicted in Figure 2. For pristine SnS_2_ (Figure 2a1–a4), the bandgap (E_g_) remains nearly constant (~1.58 eV) across different gas environments, indicating minimal perturbation of its intrinsic electronic structure upon gas physisorption. In contrast, both Pd–SnS_2_ (Figure 2b1–b4) and Pt–SnS_2_ (Figure 2c1–c4) exhibit significant bandgap narrowing, with E_9_ reduced to ~0.86–0.97 eV for Pd–SnS_2_ and ~1.11–1.32 eV for Pt–SnS_2_. Notably, the degree of bandgap reduction is strongly dependent on both the dopant species and the adsorbed gas: Pt–SnS_2_ shows the most pronounced bandgap narrowing in the presence of H_2_ (E_g_ = 1.322 eV), while Pd–SnS_2_ exhibits the smallest bandgap for CO (E_g_ = 0.869 eV). These observations suggest that metal doping not only modifies the Fermi level but also enhances orbital hybridization between the SnS_2_ host and gas molecules, thereby facilitating charge transfer and altering the semiconducting behavior.

**Figure 3:**
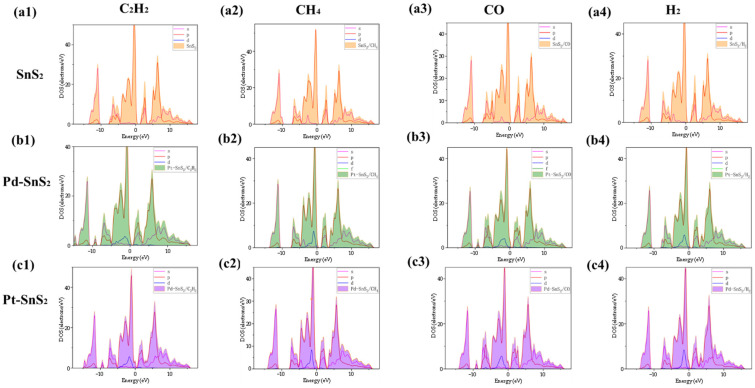
The density of status of C_2_H_2_, CH_4_, CO, and H_2_ gas molecule adsorption on pure SnS_2_ monolayers and Pd or Pt/SnS_2_ monolayers.

Figure 3 illustrates the projected density of states (PDOS) for C_2_H_2_, CH_4_, CO, and H_2_ adsorbed on pristine SnS_2_, Pd–SnS_2_, and Pt–SnS_2_ monolayers, revealing critical insights into the electronic structure modification and gas-sensing mechanisms. For pristine SnS_2_ (Figure 3a1–a4), the PDOS exhibits minimal perturbation upon gas adsorption, with valence and conduction bands retaining their intrinsic character and no significant peak shifts or new states emerging, consistent with weak physisorption. In contrast, both Pd–SnS_2_ (Figure 3b1–b4) and Pt–SnS_2_ (Figure 3c1–c4) display pronounced changes in the PDOS, particularly near the Fermi level (E_F), where new hybridized states appear due to strong orbital hybridization between the metal dopants and the adsorbed gas molecules. Notably, for Pd–SnS_2_, the PDOS for CO (Figure 3b3) exhibits the most significant redistribution, with a pronounced peak at ~−1.5 eV, indicating strong chemisorption and enhanced charge transfer. Similarly, Pt–SnS_2_ shows distinct PDOS features for H_2_ (Figure 3c4), with a noticeable shift in the d-band center, suggesting efficient catalytic dissociation. The emergence of metal–gas hybrid states below E_F in doped systems confirms the activation of gas molecules via charge transfer, which is essential for achieving high sensitivity in transformer-oil-dissolved gas detection.

**Figure 4:**
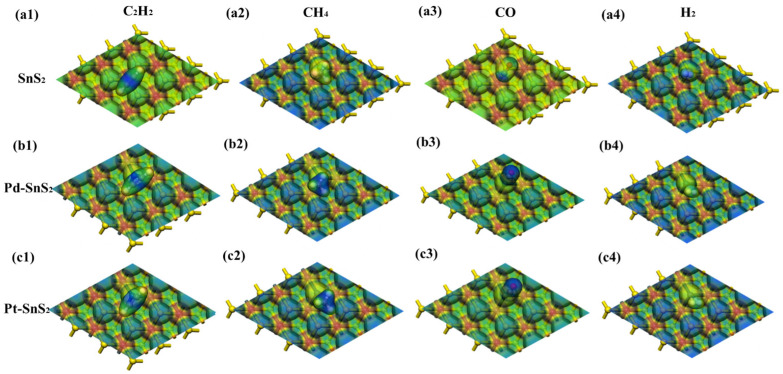
The electrostatic potential of C_2_H_2_, CH_4_, CO, and H_2_ gas molecule adsorption on pure SnS_2_ monolayers and Pd or Pt/SnS_2_ monolayers.

As seen in Figure 4, orange regions indicate electron accumulation, followed by green regions, while blue represents electron depletion. In the C_2_H_2_ system, a distinct electron-enrichment region (highlighted in green) forms between Pt and C≡C, exhibiting typical chemical adsorption characteristics. Concurrently, blue-green electron dissipation appears near the terminal hydrogen atoms, suggesting weakened hydrogen bonds during adsorption. This leads to a flattening of the molecular structure, thereby enhancing interfacial coupling. The reduction in electron density near Pt/Pd (blue regions) combined with electron aggregation around S atoms (yellow regions) constitutes a dipole rearrangement structure, facilitating interfacial charge transfer (Q_t_ = −0.29 e). In the CH_4_ system, only minor electronic perturbations were observed around the molecule with no localized density concentration zones, indicating adsorption via van der Waals forces alone. Due to its tetrahedral configuration, the molecular Pt distance is relatively large (approximately 1.9 Å), resulting in significant steric hindrance that further suppresses electronic interactions. A faint yellow transition zone appears beneath the molecule at the Pd contact region, indicating adsorption dominated by van der Waals forces with minimal electronic rearrangement. Furthermore, the tetrahedral structure of CH_4_ maintains a substantial spatial distance from the Pd site, significantly suppressing effective orbital overlap. This aligns with its low adsorption energy and charge transfer quantity. In CO adsorption, a narrow electronic bridge region appears between the carbon atom and Pt/Pd, revealing weak d-σ orbital hybridization behavior. The O-end electron density exhibits slight sparseness, suggesting its polar structure promotes an orientation-dependent adsorption mechanism. Despite the overall small charge transfer quantity (Q_t_ = −0.01 e), CO’s linear structure facilitates proximity to the Pt active site, thereby sustaining moderate adsorption energy. For the H_2_ system, only a small electron density accumulation is observed beneath the molecule, suggesting that the s or d orbitals of Pt/Pd may participate in d-type electron transfer processes. However, this process is energetically weak, and the adsorption behavior remains predominantly physical in nature, lacking distinct bonding characteristics.

**Figure 5:**
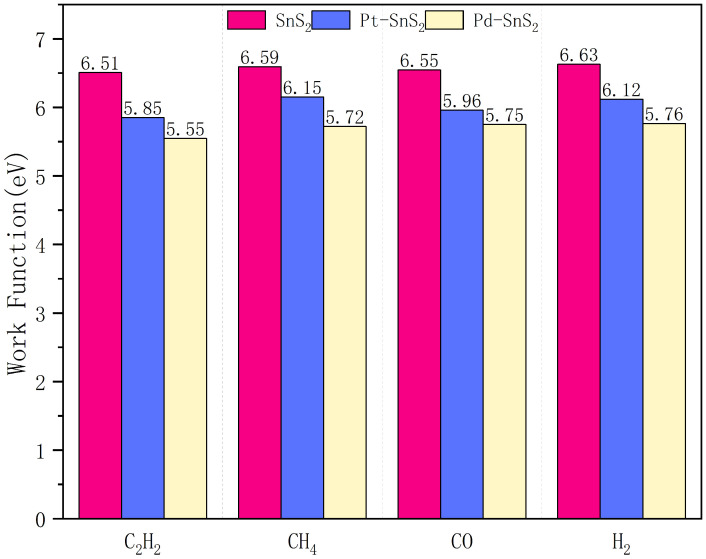
The work function of C_2_H_2_, CH_4_, CO, and H_2_ gas molecule adsorption on pure SnS_2_ monolayers and Pd or Pt/SnS_2_ monolayers.

Figure 5 presents a comparative analysis of the work functions (WFs) for pristine SnS_2_, Pt-SnS_2_, and Pd-SnS_2_ monolayers in the presence of four representative dissolved gases in transformer oil: C_2_H_2_, CH_4_, CO, and H_2_. The pristine SnS_2_ monolayer exhibits the highest WF across all gas environments, ranging from 6.51 eV (C_2_H_2_) to 6.63 eV (H_2_) and indicating its intrinsic electronic stability and relatively low reactivity toward charge transfer. In contrast, both the Pt–SnS_2_ and Pd–SnS_2_ systems show significantly reduced WFs, with Pt-SnS_2_ consistently displaying the lowest values (5.85–5.96 eV) and Pd-SnS_2_ showing intermediate values (5.55–5.76 eV). This systematic decrease in WF upon metal doping—which is particularly pronounced for Pt—suggests enhanced charge transfer from the adsorbed gas molecules to the semiconductor surface, which is a key indicator of improved gas-sensing performance. The observed trends are consistent with the modulation of the Fermi level and surface dipole moments induced by the noble metal dopants, thereby altering the Schottky barrier height and facilitating carrier transport. These findings underscore the critical role of Pt and Pd dopants in tailoring the electronic structure of SnS_2_ for selective and sensitive detection of dissolved fault gases in transformer oil.

The recovery time, a critical parameter governing the reversibility and operational stability of gas-sensing devices, was systematically investigated for SnS_2_, Pd-SnS_2_, and Pt-SnS_2_ monolayers exposed to C_2_H_2_, CH_4_, CO, and H_2_ across a temperature range of 298–498 K. In Table 2, the recovery behavior exhibits strong temperature dependence and material specificity. For pristine SnS_2_, the recovery time remains on the order of 10^−12^ s across all temperatures and target gases, indicating ultrafast desorption kinetics and excellent reversibility, albeit with minimal gas discrimination. In stark contrast, both Pd-SnS_2_ and Pt-SnS_2_ exhibit significantly prolonged recovery times, particularly at lower temperatures (298 K), reflecting stronger chemisorption interactions induced by metal doping. Notably, Pd-SnS_2_ demonstrates the most dramatic retardation in recovery for CO- and O-containing species; for instance, the recovery time for CO at 298 K reaches 1.16 × 10^9^ s, which is orders of magnitude longer than that of SnS_2_, which underscores its high selectivity and strong binding affinity toward polar or oxygenated gases. Pt-SnS_2_, exhibiting slightly faster recovery than Pd-SnS_2_ for most gases, still maintains substantially longer recovery times than pristine SnS_2_, especially for H_2_- and O-containing species at 298 K. As the temperature increases to 398 K and 498 K, the recovery times for all doped systems decrease markedly, following an Arrhenius-type trend, which suggests that thermally activated desorption processes dominate the recovery mechanism. These findings highlight a fundamental trade-off between sensitivity (favored by strong chemisorption and long recovery times) and operational speed (favored by weak physisorption and short recovery times), providing crucial guidance for optimizing metal-doped SnS_2_-based sensors for specific application scenarios under varying thermal conditions.

## 3. Calculation Method

In this work, all first-principles calculations were carried out using the CASTEP software package (Materials Studio 2020) [24]. The electron–atom interactions are described using the projector augmented-wave (PAW) potential [25,26]. The PBE pseudopotential in the generalized gradient approximation (GGA) was used to describe the exchange-correlation interactions between electrons [27], and the Grimme (DFT-D^3^) algorithm was used to optimize the van der Waals forces between the WSe_2_ monolayer and gas molecules [28]. A cut-off energy of 500 eV was used in the calculations. In addition, a 20 Å vacuum layer was set up to prevent interactions between neighboring layers. The Monkhorst–Pack k-points set in Brillouin (BZ) were 5 × 5 × 1. During structural optimization, the energy convergence value was set to 10^−6^ eV, and the convergence criterion for the force was set to 0.01 eV/Å^−1^. The initial adsorption distance was set to 3.0 Å for all systems considered attractive [29].

We calculated the adsorption energy (*Ea*) of adsorbed systems, which was defined as shown in Equation (1):

*Ea* = *E*_SnS2 + *gas molecule*_ − (*E*_SnS2_ + *E*_*gas molecule*_) (1)

where *E*_SnS2_*_ + gas molecule_* is the total energy of the Pd- and Pt-doped SnS_2_ monolayer adsorbed onto the system, *E*_SnS2_ is the energy of the Pd- and Pt-doped SnS_2_ monolayer, and *E_gas molecule_* is the energy of a gas molecule. All energies were calculated for optimized atomic structures.

## 4. Conclusions

In this paper, based on DFT and employing first-principles calculations, we systematically analyzed the adsorption of four commonly dissolved gases in transformer oils onto pure SnS_2_ and Pt, Pd-SnS_2_ monolayers. The following conclusions were drawn: Pure SnS_2_ exhibits only physical adsorption via weak van der Waals forces due to its stable surface electronic structure and scarcity of active sites, demonstrating an insufficient response to critical fault gases such as C_2_H_2_ and CO. The introduction of Pt and Pd atomic doping reconfigures the SnS_2_ electronic structure, activates surface active sites, and significantly enhances adsorption performance. Pt doping markedly enhances C_2_H_2_ adsorption: the absolute adsorption energy increases to 0.718 eV; charge transfer reaches −0.29 e; adsorption distance shortens to 1.984 Å, and localized states appear at the conduction band edge, confirming chemisorption dominance. Pd doping optimized CO selectivity: the absolute value of CO adsorption energy reached its highest at 1.246 eV; the bandgap decreased to 0.869 eV, and the work function reduced to 5.55 eV. The weak adsorption characteristics of CH_4_ and H_2_ are as follows: post-doping, the absolute adsorption energies remain below 0.521 eV (CH_4_) and 0.165 eV (H_2_), with adsorption distances exceeding 1.9 Å, indicating that doped SnS_2_ still lacks an effective response to non-polar/small-molecule gases.

In summary, Pt/Pd doping enhances the adsorption capacity of SnS_2_ towards C_2_H_2_ and CO in transformer oil by optimizing its electronic structure and surface active sites while maintaining a weak response to CH_4_/H_2_. This provides crucial theoretical support for developing highly selective gas sensors. The conclusions presented herein provide theoretical and technical support for developing Pt/Pd-SnS_2_ nanosensors for online monitoring of dissolved gases in oil.

## Figures and Tables

**Table 1:** Adsorption energy (E_ads_), electron transfer (Q_t_) and adsorption distance (D) for CH_4_, C_2_H_2_, H_2_ and CO gases adsorbed on Pd or Pt-SnS_2_ monolayer.

Systems		E_ads_ (eV)	Q_t_ (e)	D (Å)
C_2_H_2_	SnS_2_	−0.007	−0.090	2.918
	Pd-SnS_2_	−0.980	−0.260	2.130
	Pt-SnS_2_	−0.718	−0.290	1.984
CH_4_	SnS_2_	−0.027	−0.060	0.892
	Pd-SnS_2_	−0.521	−0.230	1.905
	Pt-SnS_2_	−0.099	−0.180	1.909
CO	SnS_2_	−0.005	0	1.280
	Pd-SnS_2_	−1.246	−0.010	1.733
	Pt-SnS_2_	−0.866	−0.010	1.704
H_2_	SnS_2_	−0.008	−0.050	0.974
	Pd-SnS_2_	−0.111	−0.140	1.397
	Pt-SnS_2_	−0.165	−0.170	1.387

**Table 2:** The recovery time of C_2_H_2_, CH_4_, CO, and H_2_ gas molecule adsorption on pure SnS_2_ monolayers and Pd or Pt/SnS_2_ monolayers.

Systems		298 K	398 K	498 K
CH_4_	SnS_2_	1.31 × 10^−12^	1.23 × 10^−12^	1.18 × 10^−12^
	Pd/SnS_2_	3.70 × 10^4^	2.54	8.21 × 10^−3^
	Pt/SnS_2_	1.38	1.23 × 10^−3^	1.84 × 10^−4^
C_2_H_2_	SnS_2_	2.86 × 10^−12^	2.20 × 10^−12^	1.88 × 10^−12^
	Pd/SnS_2_	6.43 × 10^−4^	3.94 × 10^−6^	1.87 × 10^−7^
	Pt/SnS_2_	4.72 × 10^−11^	1.79 × 10^−11^	1.01 × 10^−11^
CO	SnS_2_	1.21 × 10^−12^	1.16 × 10^−12^	1.12 × 10^−12^
	Pd/SnS_2_	1.16 × 10^9^	5.93 × 10^3^	4.03
	Pt/SnS_2_	4.38 × 10^2^	9.17 × 10^−2^	5.77 × 10^−4^
H_2_	SnS_2_	1.37 × 10^−12^	1.26 × 10^−12^	1.20 × 10^−12^
	Pd/SnS_2_	7.53 × 10^−11^	2.54 × 10^−11^	1.33 × 10^−11^
	Pt/SnS_2_	6.16 × 10^−10^	1.23 × 10^−10^	4.67 × 10^−11^

## Data Availability

The original contributions presented in this study are included in the article. Further inquiries can be directed to the corresponding author.
